# Cognitive Impairment in Neurological Diseases

**DOI:** 10.3390/ijms25084435

**Published:** 2024-04-18

**Authors:** Julián Benito-León, Vasileios Papaliagkas

**Affiliations:** 1 Department of Neurology, University Hospital “12 de Octubre”, ES-28041 Madrid, Spain; 2 Centro de Investigación Biomédica en Red Sobre Enfermedades Neurodegenerativas (CIBERNED), ES-28041 Madrid, Spain; 3 Instituto de Investigación Sanitaria Hospital 12 de Octubre (imas12), ES-28041 Madrid, Spain; 4 Department of Medicine, Faculty of Medicine, Complutense University, ES-28041 Madrid, Spain; 5 Department of Biomedical Sciences, School of Health Sciences International Hellenic University, 57400 Thessaloniki, Greece

## 1. Introduction

The complex link between cognitive impairment and neurological disorders underscores the intricacies of neurological sciences. Recent advancements have illuminated this intersection and propelled our understanding into new territories, revealing the genetic, molecular, and environmental factors contributing to cognitive decline.

This Special Issue of the *International Journal of Molecular Sciences* aims to shed light on recent advancements and provide comprehensive reviews of specific aspects of cognitive impairment in neurological diseases. It focuses on delineating and analyzing the patterns of cognitive impairment observed across various neurological disorders such as Alzheimer’s disease, frontotemporal dementia, stroke, and amyotrophic lateral sclerosis (ALS), exploring their molecular biology, pathophysiology, and diagnostic methods. This edition includes studies involving both animal models and human subjects.

The issue features five articles that underscore the global scientific community’s dedication to understanding the complex relationships involved in cognitive impairment associated with neurological diseases. Two of these studies provide significant insights into experimental and methodological approaches for researching cognitive impairment using animal models. The remaining articles focus on the development of early and precise diagnostic techniques for cognitive impairment in Alzheimer’s disease and other neurodegenerative disorders. These include advanced methods involving cerebrospinal fluid (CSF) and plasma biomarkers alongside a comprehensive suite of neuropsychological evaluations.

## 2. Exploring Biomarkers in Neurological Disease Diagnostics

In a meticulously designed study by Mastrangelo et al. [1], an increase in plasma glial fibrillary acidic protein levels was observed in ALS patients compared to controls. This elevation was primarily attributed to concurrent Alzheimer’s disease pathology, suggesting its potential as a reliable biomarker for cognitive impairment in ALS. As we delve deeper into the molecular underpinnings of cognitive impairment, it becomes evident that neurological conditions, including ALS and Alzheimer’s disease, might be more interconnected than previously thought [2]. This work enriches our understanding of the disease mechanisms and opens the door to novel therapeutic targets, potentially revolutionizing treatment paradigms and offering hope for patients grappling with these debilitating conditions.

Neuropsychometric tests and CSF protein analyses are routinely employed in clinical settings to assess dementia patients and are integral to disease diagnosis. The most commonly used CSF diagnostic biomarkers for Alzheimer’s disease include β-amyloid, total tau protein, and phospho-tau protein, though more recent biomarkers are also under investigation [3]. Two other articles in this special issue further elaborate on this topic. One discusses the current state of our knowledge regarding CSF biomarkers in diagnosing Alzheimer’s disease, while the other examines diagnostic biomarkers for assessing cognitive impairment in patients with the behavioral variant of frontotemporal dementia.

## 3. Technological Frontiers in Diagnosis and Treatment

The advent of cutting-edge technological advancements promises to redefine the landscape of diagnostic and therapeutic strategies in cognitive impairment. From sophisticated neuroimaging techniques that offer unprecedented views of the brain’s structure and function to the discovery of biomarkers that facilitate early detection and intervention, these innovations herald a new era in neurological care. The move towards personalized medicine, where treatments are tailored to each patient’s unique genetic makeup and disease manifestation, underscores the complexity of cognitive impairment across neurological disorders. It challenges us to rethink our approaches and advocate for treatments that address the individual, not just the illness.

## 4. The Essential Tremor–Cognition Nexus: A Case Study

Exploring the nexus between essential tremor (ET) and cognitive impairment is a paradigmatic case study within this broader inquiry into the cognitive landscape of neurological disorders. Traditionally viewed as a primarily motor system disorder characterized by its tremulous manifestations, ET has recently been scrutinized under the lens of cognitive science, revealing significant overlaps with cognitive impairments [3,4,5].

Recent research has begun to dismantle the simplistic categorization of ET as merely a motor disorder, illuminating how it intersects with cognitive functions. Studies have shown that individuals with ET are at an elevated risk of developing cognitive deficits, including but not limited to executive dysfunction, memory impairments, and difficulties with attention and processing speed [4]. These cognitive challenges can profoundly affect daily functioning and health-related quality of life, underscoring the importance of viewing ET through a holistic lens that accounts for both motor and cognitive symptoms [5,6,7].

The revelation that ET may have a significant cognitive dimension invites reevaluating diagnostic criteria and treatment paradigms. It suggests that clinicians and researchers should adopt a more comprehensive approach to understanding and managing ET, which incorporates cognitive screening and interventions as integral components of care. Moreover, this emerging perspective on ET as a neurodegenerative disorder with both motor and cognitive manifestations aligns with current trends in neuroscience that emphasize the interconnectedness of brain systems.

In light of these findings, the ET–cognition nexus represents a critical area for future research. Investigating the underlying mechanisms that link ET to cognitive impairment is essential for developing targeted therapeutic strategies that address the full spectrum of the disorder. Furthermore, understanding the relationship between ET and cognitive functions could provide valuable insights into the broader principles governing the interaction between motor disorders and cognition, with potential implications for various neurological conditions.

## 5. Patient-Centered Care: A Paradigm Shift

Amid scientific and technological advancements, the shift towards patient-centered care represents a fundamental reorientation of our treatment and care philosophies. Recognizing the profound effects of cognitive impairment on individuals’ lives necessitates approaches that go beyond addressing the biological aspects of disorders. Instead, there is a burgeoning demand for strategies that enhance patients’ overall well-being, considering their emotional, social, and psychological needs and physical health.

## 6. Looking Forward: Challenges and Opportunities

The path forward is laden with challenges and opportunities as we stand on the precipice of discoveries. The promise of unraveling the complexities of cognitive impairment in neurological disorders is contingent upon our ability to conduct longitudinal studies, integrate cutting-edge technologies, and foster interdisciplinary collaboration. The research highlighted in the Special Issue lays the groundwork for this journey, offering insights and raising questions to fuel future inquiry.

## 7. Conclusions

Navigating neurological disorders’ cognitive landscape is marked by continuous learning and adaptation. We trust that the contributions in the *International Journal of Molecular Sciences* Special Issue on “Cognitive Impairment in Neurological Diseases” will offer valuable insights into the evaluation of cognitive impairments across various neurological conditions. Additionally, we hope it will inspire new research avenues and stimulate innovative ideas among our readers and the scientific community at large. As we forge ahead, the spirit of collaboration, innovation, and patient-centered care will be pivotal in overcoming the challenges and harnessing the opportunities in our quest to mitigate cognitive impairment within the vast domain of neurological sciences.
